# Poly[di-μ_2_-chlorido(μ_2_-1,3-di-4-pyridylpropane-κ^2^ *N*:*N*′)lead(II)]

**DOI:** 10.1107/S1600536809036150

**Published:** 2009-09-16

**Authors:** Zhiyong Fu, Dongpo Su, Desheng Song

**Affiliations:** a School of Chemistry and Chemical Engineering, South China University of Technology, Guangzhou, People’s Republic of China

## Abstract

The title Pb^II^ coordination polymer, [PbCl_2_(C_13_H_14_N_2_)], was prepared by the hydro­thermal reaction of PbCl_2_ with 4,4,-trimethyl­enedipyridine in a 1:1 ratio. It exhibits a two-dimensional layered structural motif consisting of PbCl_2_ chains and the flexible bridged 4,4′-trimethyl­enedipyridine ligand. The connections result in a cavity of about 4 × 15 Å.

## Related literature

For crystal engineering based upon transition metal coordination polymers, see: Abrahams *et al.* (1999 ▶). For applications of these metal-organic frameworks, see: Moulton & Zaworotko (2001 ▶); Natarajan & Mahata (2009 ▶). For networks with main group metals as connected nodes, see: Shi *et al.* (2002 ▶). For the related structure, [PbCl_2_(4,4′-bipy)] (bipy is bipyridine), see: Nordell *et al.* (2004 ▶).
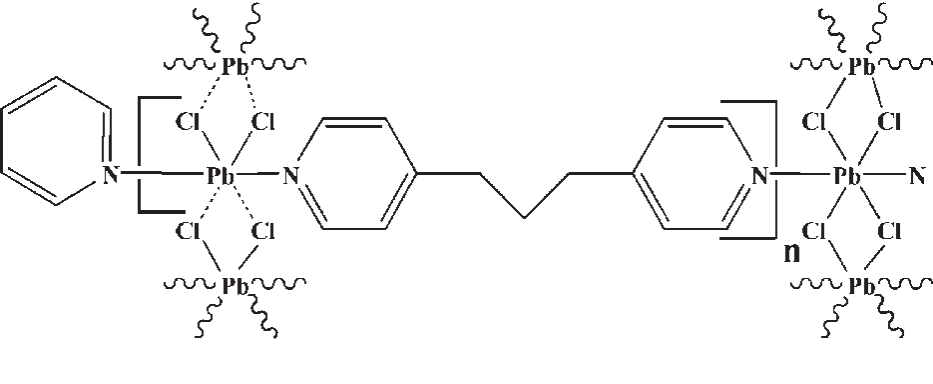


## Experimental

### 

#### Crystal data

[PbCl_2_(C_13_H_14_N_2_)]
*M* *_r_* = 476.35
Monoclinic, 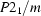

*a* = 4.385 (2) Å
*b* = 15.455 (3) Å
*c* = 10.935 (2) Å
β = 97.65 (2)°
*V* = 734.5 (3) Å^3^
*Z* = 2
Mo *K*α radiation
μ = 11.84 mm^−1^
*T* = 298 K
0.19 × 0.15 × 0.11 mm

#### Data collection

Bruker SMART CCD diffractometer
Absorption correction: multi-scan (*SADABS*; Sheldrick, 1996 ▶) *T*_min_ = 0.139, *T*_max_ = 0.277
2401 measured reflections
1283 independent reflections
1109 reflections with *I* > 2σ(*I*)
*R*_int_ = 0.033

#### Refinement

*R*[*F* ^2^ > 2σ(*F* ^2^)] = 0.033
*wR*(*F* ^2^) = 0.077
*S* = 1.01
1283 reflections
88 parameters
H-atom parameters constrained
Δρ_max_ = 0.88 e Å^−3^
Δρ_min_ = −1.16 e Å^−3^

### 

Data collection: *SMART* (Bruker, 1996 ▶); cell refinement: *SMART* and *SAINT* (Bruker, 1996 ▶); data reduction: *SHELXTL* (Sheldrick, 2008 ▶); program(s) used to solve structure: *SHELXS97* (Sheldrick, 2008 ▶); program(s) used to refine structure: *SHELXL97* (Sheldrick, 2008 ▶); molecular graphics: *SHELXTL*; software used to prepare material for publication: *SHELXTL*.

## Supplementary Material

Crystal structure: contains datablocks global, I. DOI: 10.1107/S1600536809036150/pb2006sup1.cif

Structure factors: contains datablocks I. DOI: 10.1107/S1600536809036150/pb2006Isup2.hkl

Additional supplementary materials:  crystallographic information; 3D view; checkCIF report

## Figures and Tables

**Fig. 1.**
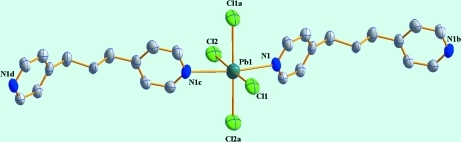
The molecular structure of (I), with atom labels and 50% probability displacement ellipsoids for non-H atoms.

**Fig. 2.**
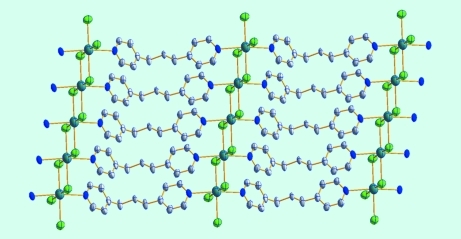
The packing of (I), viewed down the c axis.

## supplementary crystallographic information

### Comment

Crystal engineering based upon transition metal coordination polymers has made rapid progress (Abrahams *et al.*, 1999). These metal organic frameworks attracted much attention in the field of host guest chemistry (Natarajan *et al.*, 2009), which may find applications in catalysis, nonlinear optics, magnetism, molecular recognition and separation (Moulton *et al.*, 2001). By comparison, the networks with main group metals as connected nodes have not been well documented (Shi *et al.*, 2002). Recently, many lead halides based coordination polymers with nitrogen-containing ligand as bridge exhibit interesting physical properties and structural motifs (Nordell *et al.*, 2004). Different linkers, such as 4,4,-bipy, pyrazine and bipyridyl-based butadiene are introduced to the construction of lead halide organic-inorganic hybrid compounds. Here we report the hydrothermal synthesis and structural characterization of a new coordination complex based on PbCl_2_ inorganic unit and 4,4,-trimethylenedipyridine. Hydrothermal reaction of PbCl_2_ and 4,4,-trimethylenedipyridine with equimolar amounts afford block-like crystals. They were characterized by single-crystal X-ray structural analysis. Details of crystallographic data for the title compounds 1 is listed in Table 1. The structure of PbCl2(4,4,-trimethylenedipyridine) framework is a two-dimensional-layered motif constructed by the [PbCl_2_]_n_ chains and the flexible bridge 4,4,-trimethylenedipyridine ligand (Fig. 1). The crystal is monoclinic, space group P21/m, with the Pb, Cl1 and Cl2 atoms lying on a crystallographic mirror plane. Each lead metal center is six-coordinate geometry with four chloride ion on the square plane and two nitrogen donors at the axial direction. The bond distances of Pb—Cl range from 2.862 (6) Å to 2.982 (6) Å. And the bond distance of Pb—N is 2.667 (7) Å. These parameters are close to previous report (Nordell *et al.*, 2004). The bond angles of Cl—Pb—Cl at the square plane vary from 81.15 (17) to 97.21 (17)°. And the *trans* N1—Pb1—N1 bond angle is 166.1 (3)°. These value indicate that the lead center is situated in a distorted octahedral environment and the lone pair in Pb(II) is stereochemically active. As showed in figure 2, The [PbCl_2_]_n_ chains are linked into flat sheets by the 4,4,-trimethylenedipyridine bridges. The dimensions of the distorted square cavity are approximately 4*15 Å. The flexible of the spacers make the layer into an undulating structural motif. And the sheets stack along *a* axis at a distance of 4.69 Å.

### Experimental

An aqueous mixture (10 ml) containing 4,4,-trimethylenedipyridine (0.1 g, 0.5 mmol), PbCl_2_ (0.139 g, 0.5 mmol) was placed in a Parr Teflonlined stainless steel vessel (25 ml), and the vessel was sealed and heated to 403.15 K for 24 h. 0.08 g block-like crystals were obtained.

### Refinement

H atoms were positioned geometrically and refined using a riding model, with C—H = 0.93–0.97 Å and with *U*_iso_(H) = 1.2 (1.5 for methyl groups) times *U*_eq_(C). The non-hydrogen atoms were refined anisotropically. 41 low-theta reflections were omitted from the data set.

### Crystal data

**Table:**

[PbCl_2_(C_13_H_14_N_2_)]	*F*(000) = 444
*M**_r_* = 476.35	*D*_x_ = 2.155 Mg m^−^^3^
Monoclinic, *P*2_1_/*m*	Melting point: 533.15K K
Hall symbol: -P2yb	Mo *K*α radiation, λ = 0.71073 Å
*a* = 4.385 (2) Å	Cell parameters from 1283 reflections
*b* = 15.455 (3) Å	θ = 2.6–25.0°
*c* = 10.935 (2) Å	µ = 11.84 mm^−^^1^
β = 97.65 (2)°	*T* = 298 K
*V* = 734.5 (3) Å^3^	Block, yellow
*Z* = 2	0.19 × 0.15 × 0.11 mm

### Data collection

**Table:**

Bruker SMART CCD diffractometer	1283 independent reflections
Radiation source: fine-focus sealed tube	1109 reflections with *I* > 2σ(*I*)
graphite	*R*_int_ = 0.033
ω scans	θ_max_ = 25.0°, θ_min_ = 2.6°
Absorption correction: multi-scan (*SADABS*; Sheldrick, 1996)	*h* = −5→5
*T*_min_ = 0.139, *T*_max_ = 0.277	*k* = −15→18
2401 measured reflections	*l* = −7→12

### Refinement

**Table:**

Refinement on *F*^2^	Primary atom site location: structure-invariant direct methods
Least-squares matrix: full	Secondary atom site location: difference Fourier map
*R*[*F*^2^ > 2σ(*F*^2^)] = 0.033	Hydrogen site location: inferred from neighbouring sites
*wR*(*F*^2^) = 0.077	H-atom parameters constrained
*S* = 1.01	*w* = 1/[σ^2^(*F*_o_^2^) + (0.0359*P*)^2^] where *P* = (*F*_o_^2^ + 2*F*_c_^2^)/3
1283 reflections	(Δ/σ)_max_ < 0.001
88 parameters	Δρ_max_ = 0.88 e Å^−^^3^
0 restraints	Δρ_min_ = −1.16 e Å^−^^3^

### Special details

**Table:**

Geometry. All e.s.d.'s (except the e.s.d. in the dihedral angle between two l.s. planes) are estimated using the full covariance matrix. The cell e.s.d.'s are taken into account individually in the estimation of e.s.d.'s in distances, angles and torsion angles; correlations between e.s.d.'s in cell parameters are only used when they are defined by crystal symmetry. An approximate (isotropic) treatment of cell e.s.d.'s is used for estimating e.s.d.'s involving l.s. planes.
Refinement. Refinement of *F*^2^ against ALL reflections. The weighted *R*-factor *wR* and goodness of fit *S* are based on *F*^2^, conventional *R*-factors *R* are based on *F*, with *F* set to zero for negative *F*^2^. The threshold expression of *F*^2^ > σ(*F*^2^) is used only for calculating *R*-factors(gt) *etc*. and is not relevant to the choice of reflections for refinement. *R*-factors based on *F*^2^ are statistically about twice as large as those based on *F*, and *R*- factors based on ALL data will be even larger.

### Fractional atomic coordinates and isotropic or equivalent isotropic displacement parameters (Å^2^)

**Table:**

	*x*	*y*	*z*	*U*_iso_*/*U*_eq_	
Pb1	0.32077 (9)	0.7500	0.28349 (4)	0.03780 (17)	
Cl2	0.7798 (10)	0.7500	0.1052 (3)	0.0748 (10)	
Cl1	−0.1094 (10)	0.7500	0.4619 (3)	0.0719 (10)	
C5	0.6217 (18)	0.4150 (5)	0.2292 (8)	0.0431 (19)	
N1	0.3822 (16)	0.5787 (4)	0.2702 (7)	0.0482 (17)	
C2	0.412 (2)	0.4554 (5)	0.1409 (8)	0.049 (2)	
H2A	0.3481	0.4278	0.0662	0.059*	
C3	0.574 (2)	0.5401 (5)	0.3555 (8)	0.057 (2)	
H3A	0.6288	0.5684	0.4303	0.068*	
C1	0.297 (2)	0.5358 (5)	0.1631 (8)	0.049 (2)	
H1A	0.1573	0.5616	0.1026	0.059*	
C6	0.770 (2)	0.3312 (5)	0.2033 (9)	0.055 (2)	
H6A	0.9711	0.3283	0.2527	0.066*	
H6B	0.8024	0.3302	0.1171	0.066*	
C31	0.698 (2)	0.4595 (5)	0.3397 (9)	0.055 (2)	
H3B	0.8335	0.4349	0.4030	0.066*	
C7	0.584 (2)	0.2500	0.2302 (11)	0.040 (3)	
H7A	0.3863	0.2500	0.1783	0.048*	
H7C	0.5474	0.2500	0.3159	0.048*	

### Atomic displacement parameters (Å^2^)

**Table:**

	*U*^11^	*U*^22^	*U*^33^	*U*^12^	*U*^13^	*U*^23^
Pb1	0.0342 (2)	0.0274 (2)	0.0527 (3)	0.000	0.00873 (17)	0.000
Cl2	0.095 (3)	0.082 (2)	0.047 (2)	0.000	0.0061 (17)	0.000
Cl1	0.102 (3)	0.065 (2)	0.048 (2)	0.000	0.0045 (18)	0.000
C5	0.043 (5)	0.026 (4)	0.066 (6)	−0.005 (3)	0.027 (4)	0.002 (4)
N1	0.057 (4)	0.027 (3)	0.062 (5)	0.006 (3)	0.016 (4)	0.006 (3)
C2	0.062 (5)	0.032 (4)	0.052 (5)	−0.003 (4)	0.008 (4)	−0.003 (4)
C3	0.081 (7)	0.032 (4)	0.056 (6)	0.002 (4)	0.003 (5)	−0.001 (4)
C1	0.053 (5)	0.032 (4)	0.059 (6)	−0.002 (4)	−0.002 (4)	0.009 (4)
C6	0.056 (5)	0.032 (4)	0.083 (7)	−0.002 (4)	0.029 (5)	0.004 (4)
C31	0.060 (6)	0.040 (4)	0.066 (6)	−0.007 (4)	0.005 (5)	0.006 (4)
C7	0.035 (6)	0.023 (5)	0.064 (7)	0.000	0.012 (5)	0.000

### Geometric parameters (Å, °)

**Table:**

Pb1—N1^i^	2.667 (7)	C2—C1	1.374 (10)
Pb1—N1	2.667 (7)	C2—H2A	0.9300
Pb1—Cl2^ii^	2.862 (6)	C3—C31	1.379 (11)
Pb1—Cl1	2.887 (5)	C3—H3A	0.9300
Pb1—Cl1^iii^	2.957 (6)	C1—H1A	0.9300
Pb1—Cl2	2.982 (6)	C6—C7	1.548 (10)
Cl2—Pb1^iii^	2.862 (6)	C6—H6A	0.9700
Cl1—Pb1^ii^	2.957 (6)	C6—H6B	0.9700
C5—C31	1.392 (12)	C31—H3B	0.9300
C5—C2	1.390 (12)	C7—C6^iv^	1.548 (10)
C5—C6	1.494 (11)	C7—H7A	0.9700
N1—C3	1.313 (11)	C7—H7C	0.9700
N1—C1	1.354 (11)		
			
N1^i^—Pb1—N1	166.1 (3)	C1—C2—H2A	119.8
N1^i^—Pb1—Cl2^ii^	92.49 (16)	C5—C2—H2A	119.8
N1—Pb1—Cl2^ii^	92.49 (16)	N1—C3—C31	123.2 (8)
N1^i^—Pb1—Cl1	96.69 (14)	N1—C3—H3A	118.4
N1—Pb1—Cl1	96.69 (14)	C31—C3—H3A	118.4
Cl2^ii^—Pb1—Cl1	84.43 (17)	N1—C1—C2	122.1 (7)
N1^i^—Pb1—Cl1^iii^	87.32 (16)	N1—C1—H1A	119.0
N1—Pb1—Cl1^iii^	87.32 (16)	C2—C1—H1A	119.0
Cl2^ii^—Pb1—Cl1^iii^	178.36 (9)	C5—C6—C7	114.3 (7)
Cl1—Pb1—Cl1^iii^	97.21 (17)	C5—C6—H6A	108.7
N1^i^—Pb1—Cl2	83.26 (14)	C7—C6—H6A	108.7
N1—Pb1—Cl2	83.26 (14)	C5—C6—H6B	108.7
Cl2^ii^—Pb1—Cl2	97.21 (17)	C7—C6—H6B	108.7
Cl1—Pb1—Cl2	178.36 (11)	H6A—C6—H6B	107.6
Cl1^iii^—Pb1—Cl2	81.15 (17)	C3—C31—C5	120.1 (8)
Pb1^iii^—Cl2—Pb1	97.21 (17)	C3—C31—H3B	120.0
Pb1—Cl1—Pb1^ii^	97.21 (17)	C5—C31—H3B	120.0
C31—C5—C2	116.2 (7)	C6—C7—C6^iv^	108.3 (9)
C31—C5—C6	122.2 (8)	C6—C7—H7A	110.0
C2—C5—C6	121.5 (8)	C6^iv^—C7—H7A	110.0
C3—N1—C1	117.9 (7)	C6—C7—H7C	110.0
C3—N1—Pb1	118.2 (6)	C6^iv^—C7—H7C	110.0
C1—N1—Pb1	121.0 (5)	H7A—C7—H7C	108.4
C1—C2—C5	120.5 (8)		

Symmetry codes: (i) *x*, −*y*+3/2, *z*; (ii) *x*−1, *y*, *z*; (iii) *x*+1, *y*, *z*; (iv) *x*, −*y*+1/2, *z*.
